# Disseminated BCG infection and rotavirus enteritis in an infant with severe immunodeficiency: a causal association study

**DOI:** 10.3389/fpubh.2025.1695162

**Published:** 2025-12-05

**Authors:** Jianlin Cai, Shuping Li, Jingyi Xu, Zhen Li, Fang Wang, Liqing Yang, Bin Jia, Yunhua Bai, Li Li

**Affiliations:** Chaoyang District Center for Disease Control and Prevention, Beijing, China

**Keywords:** severe combined immunodeficiency (SCID), live attenuated vaccines, disseminated Bacillus Calmette–Guérin (BCG) infection, rotavirus enteritis, adverse events following immunization (AEFI)

## Abstract

Live attenuated vaccines may cause life-threatening complications in infants with undiagnosed severe combined immunodeficiency (SCID). We describe a fatal case of disseminated Bacillus Calmette–Guérin infection and rotavirus enteritis in an infant with SCID to evaluate vaccine–adverse event causality and discuss implications for immunization policy. A multidisciplinary team retrospectively reviewed clinical records, microbiological and immunological tests, genetic analysis, and vaccination history. Causality was assessed using the WHO-UMC system and the revised WHO classification for adverse events following immunization. The infant received BCG at birth and oral pentavalent rotavirus vaccine at 8 weeks, then developed disseminated BCG infection and persistent rotavirus enteritis. A frameshift variant in TNFRSF13B, consistent with a combined immunodeficiency phenotype, was identified; SCID was diagnosed by integrating this genetic result with the patient’s clinical and immunologic findings. Despite intensive anti-tuberculosis therapy and hematopoietic stem cell transplantation, she died at 13 months. Both BCG dissemination and rotavirus enteritis were considered vaccine-associated in the setting of an underlying, undiagnosed SCID; the SCID was not attributable to vaccination. To the best of our knowledge, this is the only case identified in Chaoyang District surveillance in which live attenuated vaccination was temporally associated with a fatal severe adverse outcome. This case underscores that live vaccines administered to neonates with unrecognized immune disorders—later confirmed as SCID—can result in fatal complications. Therefore, integrating newborn screening for severe combined immunodeficiency with comprehensive pre-vaccination assessment is essential to identify high-risk infants and tailor vaccine schedules. We propose that these approaches be evaluated in prospective pilot studies. We further suggest that public-health authorities consider pilot-testing tuberculosis surveillance and household exposure assessment as components of risk-stratified immunization strategies for vulnerable populations.

## 1 Introduction

Severe combined immunodeficiency (SCID) is a rare, inherited immunodeficiency, characterized by defective lymphocyte differentiation and profound T-and B-cell dysfunction (1–4). Without curative treatment such as hematopoietic stem cell transplantation (HSCT) or gene therapy, most affected infants die from severe infections within the first year of life (5). Early diagnosis—ideally before the onset of infection—can raise long-term survival rates above 90%, whereas HSCT outcomes are poor in the presence of active disease (6). T-cell receptor excision circle (TREC) testing enables early identification of SCID prior to irreversible complications or death (7, 8). In China, where SCID screening is not yet standard, newborns receive Bacillus Calmette–Guérin (BCG) vaccine at birth and other live vaccines, such as rotavirus vaccine (from 6 weeks), oral poliovirus vaccine (at 4 months), and measles, mumps, and rubella vaccine (at 8 months), which is before SCID is typically diagnosed, despite formal contraindications in immunocompromised infants.

The BCG vaccine, a live attenuated vaccine derived from *Mycobacterium bovis*, is widely recommended for newborns to prevent tuberculosis (TB) and remains a cornerstone of TB control in high-burden regions (9, 10). China, one of the 30 high TB-burden countries in the world, has also included BCG in its national immunization program. While BCG is effective in reducing the incidence of severe childhood TB forms—such as miliary TB and TB meningitis—it carries significant risks in immunocompromised individuals (11, 12). As a live attenuated vaccine, BCG can cause localized or disseminated infection in those with impaired cellular immunity. The estimated incidence of disseminated BCG disease is approximately 0.06 to 1.56 per million vaccines, typically occurring within 6 months of vaccination and associated with a high case-fatality rate of 80–83% (9, 13, 14).

The oral pentavalent reassortant rotavirus vaccine contains five human-bovine reassortant strains (G1, G2, G3, G4, P[1]A), achieving 70–95.5% protection against severe rotavirus enteritis (15). Although rotavirus enteritis is typically self-limiting in immunocompetent infants, immunodeficient infants may experience prolonged viral shedding and severe or chronic enteritis (16). The shared risks associated with BCG and rotavirus live-attenuated vaccines underscore the critical need for pre-vaccination immune assessment and careful benefit–risk evaluation in populations at high risk for primary immunodeficiencies.

This case demonstrates that, in the context of an undiagnosed SCID, live vaccines can act as precipitating events leading to disseminated BCG infection, rotavirus enteritis, and fatal multi-organ failure. To the best of our knowledge, within routine clinical surveillance in Chaoyang District, Beijing, this is the only case identified to date in which administration of live attenuated vaccines was temporally associated with a severe adverse outcome that culminated in death. Although similar reports are uncommon worldwide, this case underscores systemic gaps in newborn screening—especially in regions lacking tools such as TREC testing. The high fatality rate of BCG infection in SCID patients reinforces the WHO’s recommendation for risk-stratified immunization strategies. Based on the case, we advocate for adjustments to current immunization practices—prioritizing SCID screening, optimizing individualized schedules for live vaccines, and enhancing clinical awareness to prevent avoidable deaths among immunocompromised infants.

## 2 Materials and methods

### 2.1 Data sources

The data comprised the patient’s clinical records, the credentials of the vaccination centers and personnel, vaccine distribution documentation, cold chain monitoring records, informed consent forms, and other materials pertinent to the vaccination process.

### 2.2 Methods

In this retrospective study, a multidisciplinary expert panel convened by the Chaoyang District, Beijing, Suspected Adverse Events Following Immunization Investigation and Diagnosis Committee—comprising specialists in vaccinology, epidemiology, pediatric respiratory medicine, tuberculosis, and hematology—conducted a thorough evaluation and investigation. Following the Vaccine Administration Law of the People’s Republic of China, pertinent administrative regulations, national technical guidelines, and relevant WHO documents, as well as principles of evidence-based medicine, the panel reviewed: (1) the patient’s clinical presentation, including onset, diagnosis, treatment, progression, and outcome; (2) medical examinations and laboratory findings; and (3) vaccine characteristics and quality-control data. This comprehensive assessment integrated case history, pathogen and immunological testing, genetic results, and vaccination records to determine causality and inform subsequent discussion.

### 2.3 Ethics approval and consent to participate

This study is a retrospective analysis of cases identified and collected during routine surveillance for suspected adverse events following immunization (AEFI) in Chaoyang District. The analysis was performed on de-identified clinical case records, vaccination records, and vaccine lot/quality information; and no prospective interventions or clinical trial procedures were undertaken. The Ethics Committee of the Chaoyang District Center for Disease Control and Prevention (Beijing) reviewed the submitted materials and granted a waiver of informed consent (decision no. 20251106-1-01), acknowledging that all data were de-identified and obtained from routine public-health surveillance. Accordingly, the study was exempted from additional ethical review and guardian consent was waived.

## 3 Results

### 3.1 Patient information and vaccination history

A female neonate was delivered via cesarean section at 37 + 4 weeks’ gestation at a hospital in Beijing. Her Apgar scores at 1, 5, and 10 min were all 10 (reported verbatim in the delivery record). The mother’s postpartum course was uneventful, and there was no family history of allergic disorders. At 08:30 on the following morning, the infant received 0.1 mL of intradermal BCG vaccine in the left upper arm, in accordance with the national immunization schedule. At the age of 1 month and 26 days in the morning, she received her first dose of 2 mL oral pentavalent reassortant rotavirus vaccine (Vero cell) at a community health center. All vaccines were supplied through certified channels and maintained under strict cold-chain conditions. The immunization personnel held the requisite qualifications and had completed specialized training in vaccination techniques; all administration procedures adhered strictly to the Beijing Municipal Technical Guidelines for Immunization Practice.

### 3.2 Brief disease occurrence by timeline

At 1 month and 27 days of age, 1 day after receiving the oral pentavalent reassortant rotavirus vaccine, the infant developed a rash that began on the face and extremities and subsequently became generalized. On that day she developed multiple erythematous papules on the back and hands, accompanied by yellow crusting over the nasal bridge. At a Beijing pediatric hospital, impetigo was suspected; scabies testing was negative. Topical mupirocin and hydrocortisone butyrate were applied to facial and nasal lesions, alongside a traditional Chinese medicine for dermatology and medicated washes, resulting in partial improvement of nasal crusting within 1 week. Subsequently, the eruption progressed: facial papules formed vesicles that ruptured and crusted, accompanied by pruritus, while truncal lesions evolved from erythematous papules to yellow-red plaques. During the third to fourth month of life, the infant underwent comprehensive evaluation at multiple centers, including complete blood counts, serum biochemistry, lymphocyte subset analysis, abdominal ultrasonography, and skin histopathology. Findings of granulomatous dermatitis, lymphadenopathy, thymic hypoplasia, marked hypogammaglobulinemia (IgA, IgG, IgM), eosinophilia, anemia, and suspected splenic lesions prompted consideration of primary immunodeficiency.

At 4 months and 3 days of age, she was admitted to the rheumatology-immunology ward of a children’s hospital in a city in Hebei Province with fever, rash, and cough. Diagnoses included Pneumocystis jirovecii pneumonia, tuberculous pneumonia, disseminated BCG infection, primary immunodeficiency, and rotavirus enteritis. Empirical antimicrobial and anti-tubercular therapies were initiated. Owing to respiratory failure and clinical deterioration, at 5 months and 4 days of age she was transferred to a tertiary pediatric center in Beijing.

From admission until her death at 13 months of age, the infant received prolonged intensive care—first in the emergency intensive care unit, then in the immunology and stem cell transplantation services. Between 7 months and 11 days and 7 months and 19 days of age, she was temporarily managed at a general hospital for symptomatic treatment. After comprehensive evaluation integrating clinical manifestations, treatment response, and multiple laboratory findings—including complete blood count, immunoglobulin profile, flow cytometric analysis of T-, B-, and NK-cell subsets, lymphocyte proliferation assays, and helper T-cell subset profiling—the infant was definitively diagnosed with SCID. Despite aggressive anti-infective, anti-tubercular, and supportive therapies, she developed sepsis, severe pneumonia, disseminated BCG disease, and rotavirus enteritis. At 8 months and 23 days of age, she underwent allogeneic hematopoietic stem cell transplantation. Post-transplant complications included recurrent fever, polymicrobial infections, refractory septic shock, pulmonary hemorrhage, acute respiratory distress syndrome, and multi-organ failure, culminating in her death at 13 months of age.

### 3.3 Complex clinical presentation and diagnostic workup

The diagnosis of SCID was based on the infant’s early onset of recurrent rash, diarrhea with intermittent fever, failure to gain weight, and growth retardation, followed by progressive respiratory distress. Metagenomic next-generation sequencing (mNGS) of blood and bronchoalveolar lavage fluid, along with endotracheal aspirate cultures, revealed mixed infections including Pneumocystis jirovecii, *Mycobacterium tuberculosis* complex, *Mycoplasma pneumoniae*, group A rotavirus, *Aeromonas hydrophila*, *Enterobacter cloacae*, and SARS-CoV-2 Omicron BF.7, which proved refractory to antimicrobial therapy. Serial complete blood counts demonstrated sustained lymphopenia with concomitant eosinophilia. Immunological studies demonstrated marked hypogammaglobulinemia (reduced IgA, IgG, and IgM) and profound depletion of inhibitory T-cell and B-cell subsets, with detailed immunologic laboratory data presented in Table 1. Whole-exome sequencing identified a maternally inherited heterozygous frameshift variant in TNFRSF13B (c.105del, p. Glu36LysfsTer48; MAF0.0000544), classified as likely pathogenic and consistent with an immunoglobulin A deficiency type 2 and common variable immunodeficiency type 2 phenotype. Taken together, the clinical course, refractory polymicrobial infections, abnormal flow-cytometric immunophenotype (profound T- and B-cell lymphopenia and impaired lymphocyte function), and the genetic finding support a diagnosis of SCID.

**Table 1:** Immunologic profile at presentation (lymphocyte subsets and immunoglobulins).

Category	Index name	Abbreviation	Sampling time	Test results of this case	Reference interval (standardized by age)	Unit
Complete blood count	Eosinophil absolute count	EOS#	1 month 27 days after birth	1.79↑	0.07–1.02	10^9/L
	Eosinophil percentage	EOS%	1 month 27 days after birth	15.7↑	1–10	%
	Total lymphocyte count	LY#	1 month 27 days after birth	1.16↓	2.4–9.5	10^9/L
	Lymphocyte percentage	LY%	1 month 27 days after birth	10.2↓	26–83	%
Immunoglobulin series	Immunoglobulin G	IgG	3 months after birth	1.68↓	2.75–7.50	g/L
	Immunoglobulin A	IgA	3 months after birth	0.0667↓	–	g/L
	Immunoglobulin M	IgM	3 months after birth	0.45↓	–	g/L
	Total Immunoglobulin E	Total IgE	3 months after birth	>500↑	<15	IU/mL
Flow-cytometric lymphocyte subsets (CD markers)	T lymphocytes (CD3^+^ T cells)	CD3^+^T cells	3 months 3 days after birth	37.9↓	50–84	%
	Helper T lymphocytes (CD3^+^CD4^+^T cells)	CD4^+^T cells	3 months 3 days after birth	32.9↓	30–67	%
	Cytotoxic T lymphocytes (CD3^+^CD8^+^T cells)	CD8^+^T cells	3 months 3 days after birth	2.6↓	23–50	%
	B lymphocytes (CD19^+^B cells)	CD19^+^ B cells	3 months 3 days after birth	6.3↓	16.57–27.65	%
	Natural killer cells (CD3^−^CD16^+^CD56^+^)	NK cells	3 months 3 days after birth	53.8↑	4.84–15.47	%
Lymphocyte functional assay	Naïve helper T lymphocytes (CD3^+^CD4^+^CD45RA^+^ T cells)	Naïve CD4^+^ T cells	3 months 7 days after birth	1.4↓	67.52–89.73	%
	Naïve cytotoxic T lymphocytes (CD3^+^CD8^+^CD45RA^+^ T cells)	Naïve CD8^+^ T cells	3 months 7 days after birth	0↓	63.39–94.29	%
	Naïve B lymphocytes (CD19^+^IgD^+^CD27^−^ B cells)	Naïve B cells	3 months 7 days after birth	90.1	79.80–93.04	%
	Natural killer cells (CD3^−^CD16^+^CD56^+^)	NK cells	3 months 7 days after birth	43↑	4.84–15.47	%
	Naïve helper T lymphocytes (CD3^+^CD4^+^CD45RA^+^ T cells)	Naïve CD4^+^ T cells	5 months 8 days after birth	0↓	1,433–3,874	cells/ul
	Naïve cytotoxic T lymphocytes (CD3^+^CD8^+^CD45RA^+^ T cells)	Naïve CD8^+^ T cells	5 months 8 days after birth	0↓	710–1843	cells/ul
	Naïve B lymphocytes (CD19^+^IgD^+^CD27^−^ B cells)	Naïve B cells	5 months 8 days after birth	35↓	729–1,474	cells/ul
	Natural killer cells (CD3^−^CD16^+^CD56^+^)	NK cells	5 months 8 days after birth	560	243–924	cells/ul

Reference intervals are age-specific and were obtained from standard pediatric immunology references and local laboratory manuals where available; “–” indicates that no authoritative age-specific reference interval could be identified.

In light of the patient’s confirmed primary immunodeficiency, generalized lymphadenopathy, and ultrasonographic evidence of splenic lesions, the repeated detection of *Mycobacterium tuberculosis* complex by mNGS in blood and bronchoalveolar lavage fluid following BCG vaccination—further subtyped as *Mycobacterium bovis*—together with the identification of the same organism in bone marrow samples, provided definitive evidence of disseminated BCG infection.

Rotavirus enteritis was diagnosed based on the infant’s onset of recurrent diarrhea with sour-smelling stools following oral administration of the live pentavalent reassortant rotavirus vaccine, multiple positive rapid stool tests for group A rotavirus antigen, and repeated detection of group A rotavirus sequences in blood by mNGS.

Sepsis was evident from the infant’s early presentation with intermittent fever, respiratory distress, and diarrhea. On examination, breath sounds were diminished bilaterally—more pronounced on the right—with sparse coarse rales; hepatomegaly (8 cm below the right costal margin) and splenomegaly (9 cm below the left costal margin) were present, along with hypoactive bowel sounds (0–1 sounds/min) and hypotonia. Laboratory studies revealed leukocytosis peaking at 25.06 × 10^9^/L with neutrophil predominance, procalcitonin exceeding 100 ng/mL, and markedly elevated C-reactive protein. mNGS of bronchoalveolar lavage fluid and endotracheal aspirate cultures identified mixed pathogens: Pneumocystis jirovecii, *Mycobacterium tuberculosis* complex, *Mycoplasma pneumoniae*, group A rotavirus, *Aeromonas hydrophila*, *Enterobacter cloacae*, and SARS-CoV-2 Omicron BF.7. Abdominal ultrasonography demonstrated multiple poorly defined, hypoechoic lesions in the splenic parenchyma, consistent with infectious foci.

The diagnosis of severe acute respiratory distress syndrome was made based on the clinical presentation of tachypnea and progressive hypoxemia unresponsive to nasal cannula oxygen therapy. The infant required endotracheal intubation and mechanical ventilation with a fraction of inspired oxygen (FiO_2_) of 80% and a positive end-expiratory pressure of 8 cmH_2_O. Arterial blood gas analysis showed a PaO_2_/FiO_2_ (P/F) ratio of 89. Chest radiography demonstrated bilateral decreased lung translucency.

The diagnosis of SCID was confirmed. Quantitative immunoglobulin testing showed a serum IgG level of 1.68 g/L, consistent with hypogammaglobulinemia.

### 3.4 Outcome

Despite aggressive multidisciplinary treatment—including broad-spectrum antimicrobials, immunomodulation, mechanical ventilation, and HSCT—the infant’s condition remained refractory. Persistent mixed infections in the setting of severe immunodeficiency ultimately led to septic shock, ARDS, and multi-organ failure, culminating in respiratory and circulatory collapse and death at 13 months of age.

### 3.5 Causality assessment

In this infant with undiagnosed SCID, disseminated BCG infection and rotavirus enteritis were classified as vaccine-associated events precipitated by an underlying immunodeficiency. The SCID itself was not vaccine-induced, and vaccination should not be regarded as the sole etiologic cause of the fatal outcome.

## 4 Discussion

This case highlights the rare but devastating complications that can occur following BCG vaccination in immunocompromised pediatric populations. The patient, who had undiagnosed SCID at the time of receiving live vaccines (BCG and oral rotavirus), subsequently developed disseminated BCG infection confirmed by microbiological isolation from multiple specimens, meeting the WHO diagnostic criteria. Concurrently, prolonged rotavirus shedding and clinical deterioration following vaccination underscored the extreme susceptibility of T-cell deficient hosts to live attenuated vaccines. In SCID, the absence of granuloma formation allows unchecked proliferation of attenuated mycobacteria, leading to multisystem involvement and high mortality (17). Although vaccine-related adverse events are rare, the co-occurrence of multiple complications in this case complicated diagnosis and emphasized the need for heightened clinical vigilance. Immunization providers should be alert to warning signs potentially indicative of SCID—such as failure to thrive and persistent or recurrent infections—and avoid administering rotavirus vaccines to at-risk infants. For clinicians, persistent ulceration, abscess, or rash at the vaccine site, as well as prolonged rotavirus infection, should prompt early immunological evaluation. Timely diagnosis and intervention are critical to reducing fatal outcomes in such vulnerable populations.

The patient’s clinical manifestations and laboratory findings provided strong evidence of an underlying immune disorder. The progression from localized rash to disseminated granulomatous dermatitis, lymphadenopathy, and multisystem infections was closely associated with marked hypogammaglobulinemia (IgA, IgG, and IgM) and profound depletion of both T- and B-lymphocyte subsets. Whole-exome sequencing identified a pathogenic heterozygous frameshift mutation in the TNFRSF13B gene (c.105del, p. Glu36LysfsTer48; MAF: 0.0000544), maternally inherited, confirming a molecular diagnosis of common variable immunodeficiency type 2 with associated IgA deficiency. However, TNFRSF13B variants are most often associated with antibody-deficiency phenotypes (e.g., CVID), and a single heterozygous frameshift is unlikely by itself to account for a classical SCID presentation. Without comprehensive trio sequencing or functional validation to exclude additional pathogenic variants, definitive molecular attribution is limited, and the diagnostic weighting therefore rests primarily on the clinical phenotype and immunologic profile. This genetic finding directly implicated the underlying immunodeficiency as the root cause of the disseminated BCG infection, persistent rotavirus enteritis, and concurrent opportunistic infections.

BCG vaccination policies worldwide are tailored to regional tuberculosis burden and immunodeficiency risk. High-incidence countries generally adopt universal neonatal BCG immunization, whereas low-incidence settings target only high-risk infants. Both the World Health Organization and the European Medicines Agency recommend a risk-based approach, incorporating clinical evaluation, family history, and focused laboratory screening. For instance, the United States mandates SCID screening in its newborn panel and reserves BCG for selected high-risk groups (1), while the United Kingdom performs universal perinatal HIV exposure assessment and genetic testing for infants with suggestive family or clinical indicators (18). These strategies exemplify the balance between maximizing vaccine-derived protection and minimizing adverse events, underscoring the necessity of genetic counseling and newborn screening to identify immunodeficient infants and optimize clinical outcomes.

Recent data reveal SCID incidence higher than traditionally estimated. In China Taiwan region, Chien et al. reported 1 in 11,821 newborns (19); meta-analyses from the United States, Sweden, and 13 additional studies indicate 1 in 58,000 (1, 20, 21); Germany reports 1 in 54,000 (22); Israel 1 in 22,500 (23); and Oman a median of 4.5 per 100,000 live births (24). These figures likely underestimate true prevalence due to absent or limited newborn screening in many regions. Early detection is critical: without diagnosis and treatment within the first 1–2 years, SCID is uniformly fatal. Infants undergoing HSCT before 3.5 months of age and free of prior infection achieve >90% long-term survival, whereas those transplanted after 3.5 months with active infection have only 50% survival (22). Initiation of prophylactic anti-tubercular therapy upon early diagnosis further mitigates the risk of disseminated mycobacterial disease (25). Given its non-negligible incidence, fatal natural history, and availability of curative treatment, SCID is an ideal candidate for universal newborn screening. Indeed, TREC assays demonstrate 100% sensitivity for SCID detection, with only exceptionally rare variants (e.g., certain T-cell developmental defects or ADA-SCID) evading detection (26).

Newborn SCID screening via TREC assays is now standard practice in several high-resource countries. The United States implemented universal TREC screening in 2010, achieving coverage across all 50 states and boosting long-term survival rates from <50 to >94% (1). The United Kingdom initiated regional pilots in 2015 and is rolling out nationwide, while Canada and Australia have similarly adopted provincial and national programs, respectively. A 2010–2011 pilot in the China Taiwan region screened over 90% of newborns, demonstrating feasibility in diverse settings. Moreover, European nations, Israel, and Brazil are validating cost-effective, rapid assays. Early identification through these programs enables pre-symptomatic intervention and has markedly reduced vaccine-related complications in SCID infants.

Deferring live attenuated vaccines can unmask underlying immunodeficiencies and avert vaccine-associated complications, as exemplified by policies in Oman (27) and Saudi Arabia, which postpone BCG until 6 months of age. A separate study also supports delaying BCG beyond 1 month for highly vulnerable neonates, including those exposed to HIV (28). However, in China—where nearly 10 million infants are born annually and some 30,000 pediatric TB cases occur each year—such a delay would create an untenable immunity gap. The 2–8 week incubation of *Mycobacterium tuberculosis* and high mortality in young children mean that unprotected infants face significant risk. Implementation would further require individualized TB-exposure assessments (household contacts, environmental risks), exceeding the capacity of primary care. Moreover, BCG efficacy wanes with age; deferral could shorten the duration of childhood protection, potentially undermining long-term control of pediatric TB.

We acknowledge that this report describes a single observational case and cannot, by itself, justify national policy change. Informed by this case, we present three hypothesis-generating proposals for prospective pilot evaluation while retaining the original operational focus. First, where laboratory, clinical and financial capacity exist, pilot newborn SCID screening using TREC assays could be evaluated to identify high-risk infants prior to routine live-vaccine administration (29). Second, strengthened prenatal and neonatal risk assessment—including systematic review of prenatal findings, structured family-history screening, and targeted early immunologic testing—could be trialed as a risk-stratified approach permitting deferral or substitution of live vaccines in infants judged at increased risk of immunodeficiency. Third, region-specific, risk-stratified immunization measures informed by local tuberculosis epidemiology—paired with targeted household TB exposure assessment (for example, Mantoux testing of mothers and close contacts in high-burden areas)—could be explored in operational pilots to assess whether such measures reduce exposure risk before infant vaccination.

Each proposal is intended for stepwise pilot evaluation rather than immediate policy adoption. We recommend that pilots include prespecified endpoints (e.g., case detection rate, time to diagnosis, vaccine-related adverse events, clinical outcomes), prospective monitoring, and concurrent health-economic and operational assessments to determine feasibility, benefit, and scalability. Broader implementation should be based on locally generated evidence and accompanied by strengthened AEFI surveillance, diagnostic capacity, and clear clinical referral pathways.

## 5 Conclusion

This fatal case underscores the urgent need to explore pre-vaccination screening and individualized immunization strategies for infants at risk of primary immunodeficiency. By integrating targeted newborn screening with structured prenatal and neonatal risk assessment, clinicians and public-health programs can prospectively evaluate whether high-risk infants can be identified earlier and vaccine schedules safely tailored. We therefore recommend that such strategies be tested in stepwise pilot implementations with prespecified outcome measures. Future work might also evaluate targeted household tuberculosis screening of mothers and close contacts as an adjunct to inform personalized BCG vaccination decisions. Wider policy adoption should await evidence from such pilots and be accompanied by strengthened AEFI surveillance and diagnostic capacity.

## Data Availability

The original contributions presented in the study are included in the article/supplementary material, further inquiries can be directed to the corresponding author.
